# Thoracic endometriosis syndrome at University of Ilorin Teaching Hospital

**DOI:** 10.7196/SARJ.2018.v24i2.201

**Published:** 2018-06-21

**Authors:** P O Adeoye, A S Adeniran, K T Adesina, O A Ige, O R Akanbi, A Imhoagene, O O K Ibrahim, GG Ezeoke

**Affiliations:** 1 Division of Thoracic and Cardiovascular Surgery, Department of Surgery, College of Health Sciences, University of Ilorin, Nigeria; 2 Department of Obstetrics and Gynaecology, College of Health Sciences, University of Ilorin, Nigeria; 3 Department of Anaesthesia, College of Health Sciences, University of Ilorin, Nigeria; 4 Department of Surgery, University of Ilorin Teaching Hospital, Nigeria; 5 Department of Obstetrics and Gynaecology, University of Ilorin Teaching Hospital, Nigeria; 6 Department of Morbid Anatomy, College of Health Sciences, University of Ilorin, Nigeria

**Keywords:** endometriosis, thoracic endometriosis, catamenial, extra-pelvic endometriosis

## Abstract

**Background:**

Endometriosis is defined as the presence of endometrial tissue (stroma and functional glands) outside the uterine cavity in women of reproductive age. Ectopic sites are frequently located in the pelvis; extrapelvic sites have been reported in the gastrointestinal tract and thoracic cavity. Thoracic manifestation of endometriosis constitutes thoracic endometriosis syndrome (TES).

**Objectives:**

To examine the presentation pattern and outcome of in the management of TES.

**Methods:**

This study is a retrospective review of medical records of patients diagnosed with endometriosis at the University of Ilorin Teaching Hospital over a 3.5-year period from January 2014 to June 2017.

**Results:**

A total of 21 patients presented with endometriosis, of whom 8 (38.1%) presented with TES. The most common variety of TES was catamenial pleural effusion (CPE) accounting for 75%, followed by catamenial chest pain (37.5%). Two patients (25%) each presented with catamenial pneumothorax and catamenial haemoptysis, while 1 (12.5%) had catamenial surgical emphysema. Closed thoracostomy tube drainage plus chemical pleurodesis was the most frequent intervention technique, accounting for 62.5%.

**Conclusion:**

TES remains an uncommon entity, despite being the most common extrapelvic manifestation of endometriosis. CPE appeared to be the most common variant of TES in our environment. Currently available treatment options need to be improved, and more use made of video-assisted thoracoscopic surgery.

## Background

Endometriosis is defined as the presence of endometrial tissue (stroma and functional glands) outside the uterine cavity. It is therefore a situation when endometrial tissue is present in ectopic locations instead of its eutopic site in the uterus, a condition first described by Maurer *et al*. ^[1]^ in 1958. It is found among women of reproductive age, with an incidence varying between 5% and 15%, as documented in the literature.^[1–5]^

The majority of ectopic sites are in the pelvic organs, with only 8.9% - 12% reported as extrapelvic in location.^[2,3]^ While the thoracic cavity is one of the more common extrapelvic sites,^[6,7]^ a report documented gastrointestinal endometriosis as the most common site (32.3%). Another site is the urinary tract (5.9%), while together, the lungs, umbilicus, abdominal scars, liver, gall bladder, pancreas, breast and extremities constitute 61.8%.^[2]^ Endometriosis of the central nervous system and the heart has also been documented.^[4]^

Thoracic manifestation in endometriosis is varied, and collectively referred to as thoracic endometriosis syndrome (TES).^[3,5,6]^ Various theories have been propounded to explain this condition.^[2–6]^ The theory of coelomic metaplasia is premised upon the common origin of endometrial and mesothelial cell from coelomic epithelium. Appropriate pathologic stimuli (probably refluxed menstrual blood) then trigger metaplastic change. The retrograde menstruation or migration theory postulates that diaphragmatic endometrial implants result from shedding of eutopic endometrial tissue through the patent fallopian tube into the pelvis, and thence into the peritoneal fluid. The physiologic hypothesis suggest that high circulating levels of prostaglandin F_2_ present during menstruation cause vasoconstriction and bronchospasm, which predisposes to alveolar rupture, hence pneumothorax. The transgenital-transdiaphragmatic passage of air theory explains how pneumothorax develops from air movement from the vagina through the fallopian tube into the peritoneum and through congenital or acquired diaphragmatic defects into the pleural cavity. Transgenital movement of air is aided by deficiency of mucus plug during menses. The concept of clockwise peritoneal circulation, starting from the pelvis and upwards through the right paracolic gutter to the right hypochondrium, may facilitate the migration theory. Lastly, microembolisation of endometrial cells into the lungs through venous or lymphatic circulation have been postulated in the metastatic theory. Though none of these theories individually completely explains the TES phenomenon, there may be interplay between the various mechanisms.

TES remains an uncommon condition, with the literature mostly documenting case reports or case series, and the manifestation is often varied. Furthermore, there is a paucity of reporting of this condition from the African continent. We therefore constituted an endometriosis study group and present a report of this rare condition from North Central Nigeria.

## Methods

We conducted a retrospective review of medical records of patients diagnosed with endometriosis at the University of Ilorin Teaching Hospital over a 3.5-year period (January 2014 - June 2017). All patients presented through either the obstetrics and gynaecology or thoracic and cardiovascular surgery department.

Identification of cases managed was from the databases of the departments as portals of entry, as well as the hospital medical records database. The index of suspicion used in identifying possible TES patients included chest symptoms and signs related to the menstrual cycle, with or without a background diagnosis of pelvic endometriosis or chronic pelvic pain. The diagnostic approach included a history and physical examination at presentation, review of gynaecological history/gynaecologist review, management of emergency needs plus sample collection, other procedures and biopsy as required and necessary supportive treatments.

Also documented were their demographic characteristics, mode of presentation and diagnosis, the treatment offered and their outcomes. Data were collected and descriptive statistics presented using Excel (Microsoft, USA).

## Results

We documented a total of 21 patients presenting with endometriosis, representing 1.27% of all gynaecological admissions during the study period. Of these, 8 patients (38.1%) presented with TES. This represented 1.2% of admissions for thoracic disorders during the study period. The characteristics of these patients are presented in Table 1. Twelve patients (57.1%) had extrapelvic presentation, of whom the TES 8 comprised 66.7%; the others were 2 gastrointestinal (GIT) (25%) and 2 umbilical (16.7%) cases (2 patients had combined TES and GIT presentation). All patients had had multiple episodes of symptomatology for TES before presentation and diagnosis at our facility.

**Table 1:** Presentation and intervention in patients with TES

			**Manifestations**			
**Patient**	**Age(yrs)**	**Parity**	**Thoracic**	**Other**	**Intervention**	**Remarks**
1	27	0	Rt CCP, CPE	Pelvic	VATS + excision of lung cyst, CTTD + chemical pleurodesis	Recurrence, repeat chemical pleurodesis
2	35	0	Rt CPE, CHp	Pelvic	CTTD, home ambulatory system	-
3	35	0	Rt CPE	Pelvic, GIT	VATS + pleural biopsy, CTTD + chemical pleurodesis	Recurrence, declined further intervention
4	38	2	Rt CPE	Pelvic, GIT	CTTD + chemical pleurodesis, laparotomy	-
5	35	0	Rt CCP, CPT, CPE	Pelvic	CTTD + chemical pleurodesis, thoracotomy + pleurectomy	Failed chemical pleurodesis
6	27	0	Rt CPE	Pelvic	CTTD + chemical pleurodesis, thoracotomy + pleurectomy	Failed chemical pleurodesis
7	14	0	CSE	Pelvic	Conservative	-
8	19	0	Rt CCP, CPT, CHp	-	Conservative	-

**TES** = thoracic endometriosis syndrome; **Rt** = right

**CCP** = catamenial chest pain; **CPE** = catamenial pleural effusion

**CPT** = catamenial pneumothorax; **CSE** = catamenial surgical emphysema

**CHp** = catamenial haemoptysis; **VATS** = video-assisted thoracoscopic surgery

**CTTD** = closed thoracostomy tube drainage; **GIT** = gastrointestinal tract

The age range for TES was 14 - 38 years (median 31, mean 28.7 (standard deviation (SD) 8.63), and 87.5% were nulliparous. The most common variety of TES among the patients was catamenial pleural effusion (CPE) in six (75%) patients; three (37.5%) had catamenial chest pain (CCP), two (25%) each presented with catamenial pneumothorax (CPT) and catamenial haemoptysis (CHp) while one (12.5%) had catamenial surgical emphysema (CSE). In addition, four (50%) of the patients had multiple thoracic manifestations, while right-sided TES occurred in seven (87.5%). The patient with CSE had bilateral diffuse thoracic, nuchal and facial surgical emphysema. Seven patients (87.5%) had concomitant extrathoracic manifestation, all involving the pelvis, of whom two (28.6%) had an additional GIT manifestation; thus 25% of all patients with TES had GIT manifestation (Table 1).

Table 2 shows that diagnosis was based on strong clinical grounds in all patients, establishing catamenial relationship to presentation. In three (37.5%) patients who had a chest tube *in situ* prior to onset of menstruation, the effluent increased in volume and became haemorrhagic with menstruation. Histological confirmation was obtained in four (50%) cases, while serum CA-125 was performed in three patients, with elevated results found in two of them.

**Table 2:** Modality of diagnosis of TES

		**Clinical**	
**Patient**	**Histological**	**presentation**	**Serum CA-125**
1	No	Yes	No
2	No	Yes	No
3	Yes	Yes	No
4	Yes	Yes	No
5	Yes	Yes	No
6	Yes	Yes	Yes
7	No	Yes	Yes
8	No	Yes	Yes

**Fig. 1:**
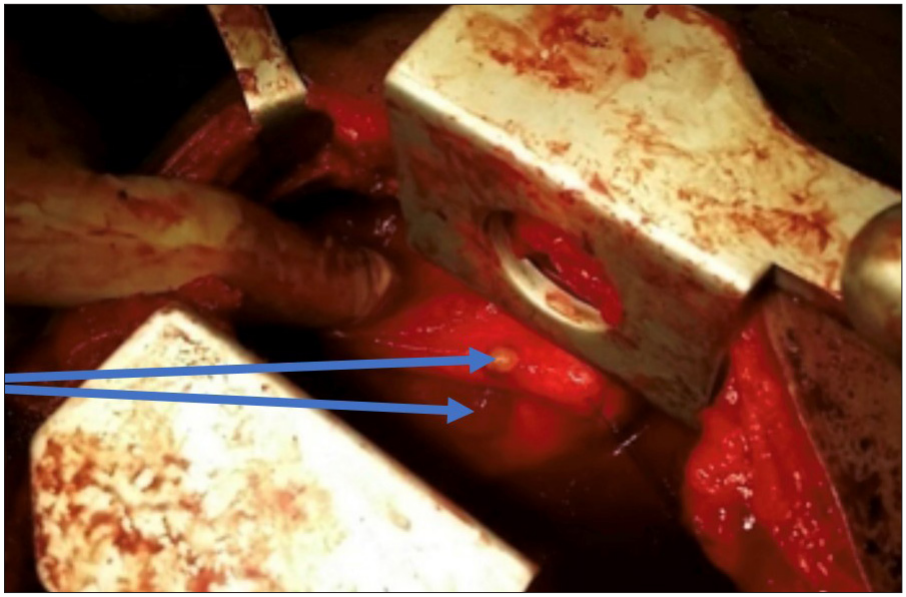
Endometriotic nodules on the diaphragm.

**Fig. 2:**
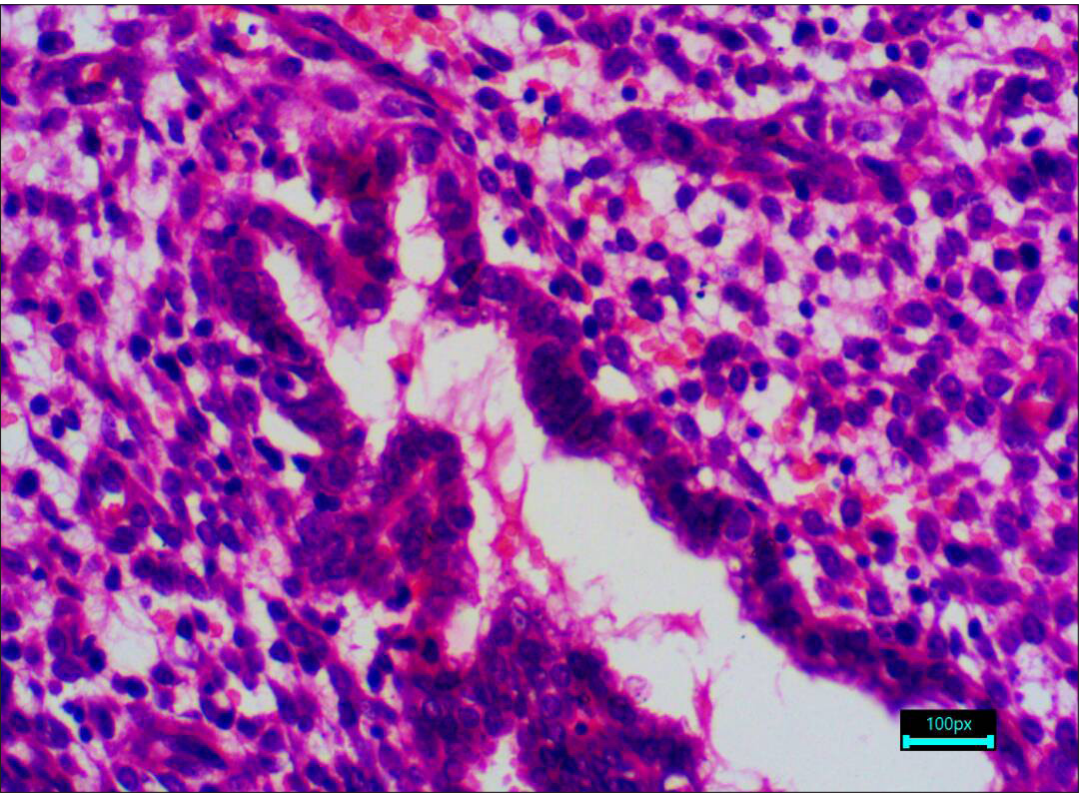
Photomicrograph of histology on endometrioma (haematoxylin and eosin staining, magnification ×400) showing a dilated endometrial gland surrounded by endometrial stroma, including mononuclear inflammatory cells (mostly macrophages).

Closed thoracostomy tube drainage (CTTD) plus chemical pleurodesis was the most frequent intervention technique, accounting for 62.5% (five patients), while diagnostic video-assisted thoracoscopic surgery (VATS) was performed on two (25%) patients. Another two (25%) had thoracotomy with parietal pleurectomy after failed chemical pleurodesis. Fig. 1 shows endometriotic nodules on the diaphragm at thoracotomy of patient 5 on the list, while Fig. 2 shows the photomicrograph of the histology of the same patient.

However, two patients were managed non-operatively. In addition, two patients had recurrent pleural effusion during subsequent menstruation following an initial successful treatment. Both were counselled for thoracotomy for parietal pleurectomy, but one opted for repeat chemical pleurodesis, while the other chose expectant management. No mortality was recorded, and patients were transferred to the gynaecology service for hormonal therapy, apart from two patients who had had hormonal treatment prior to presentation with thoracic manifestations.

## Discussion

TES refers to a constellation of manifestations resulting from growth of endometrial-like glands and stroma in the lungs, on the pleural surfaces or in the airway.^[3,5-7,9,10]^ It is an uncommon condition, documented in the literature mostly in case reports and case series.^[5-7,9-12]^ An analysis of 110 case reports/series published in English was conducted by Joseph and Sahn in 1996,^[13]^ and Channabasavaiah and Joseph,^[4]^ in 2010, conducted a similar review with the same number of patients, covering a 6.5-year period. Haga *et al*.,^[14]^ in Tokyo, Japan, reported 84 cases of CPT while evaluating 570 cases of spontaneous pneumothorax in women.Over a 7-year period, Hwang *et al*. ^[2]^ documented 15 cases at a single centre in Seoul, Korea. Forty cases were reported by Dvorakovskaya *et al*. ^[15]^ from St Petersburg Hospital, but the duration of collation was not stated. Reports from Africa are even rarer: there is a report of 3 cases by Ekpe *et al*. ^[16]^ from South South Nigeria, and a case report each from Ghana^[17]^ and Uganda. ^[18]^ There is one review article each from Nigeria and Zimbabwe. ^[3,19]^ TES is a rare but important extrapelvic manifestation of endometriosis. We therefore document these eight cases of TES seen over 3.5 years at a tertiary hospital in North Central Nigeria.

Manifestations constituting TES include CCP, CPT, CHt/CPE, catamenial haemoptysis (CHp), pulmonary nodules and CPM.^[2–8]^ These presentations result from the presence of endometrial tissue on the pleural surfaces (CCP, CPT, CPE, CPM), in the lung parenchyma (pulmonary nodule) or the airway (CHp). Pleural involvement is more common, accounting for about 83% of TES, while parenchymal and airway involvement account for 17%,^[9]^ and our study found a similar distribution. An even rarer occurrence of endometriosis on the thoracic aorta has been reported.^[20]^

The term catamenial is derived from the Greek word *Katamenios*, which means ‘monthly occurrence’. Thus development of these manifestations in a temporal relationship to menstruation is vital to clinical diagnosis. The expected interval reported between symptoms and menstruation varies, but a 72-hour period before onset and after cessation of menstruation is considered acceptable.^[13]^

Our study reflects a higher presentation of extrapelvic lesions, of 57% (12 of 21 patients with endometriosis), compared with the 8.9% - 12% documented in the literature.^[2,3]^ The higher incidence of TES (66.7%) in extrapelvic sites has been reported in some studies;^[5,6]^ however, contrary to reports of GIT dominance by others,^[2]^ in our study this constituted only 25%.

The mean age of occurrence of TES in our patients was 28.7 years (SD 8.63); this is younger than the ~35 years reported in the literature.^[2-4,8,10,13,21]^ The presentation of CHp in one of our youngest patients (19 years) may support the postulate that this manifests at a relatively younger age than other forms of manifestation.^[4]^

An interesting finding from this study is the predominance of CPE, accounting for 75% of cases, while CCP was second, with 37.5%. We also identified a rare occurrence of CSE without associated pneumothorax. Most reports present either CPT (up to 73%)^[3–7]^ or CCP (80% - 90%)^[3,7,21]^ as the most common manifestation of TES. In a review of 15 cases by Hwang *et al*.,^[2]^ CHt accounted for 53%, while the remaining 47% were CPT cases. The 87.5% predominance on the right side in this study is consistent with previous reports of the vast majority of TES (above 85%) occurring in the right hemithorax.^[2,3,5,6,10,13,21]^ This predilection has been attributed to clockwise peritoneal circulation by some authors.^[5]^

Multisite involvement in extragenital endometriosis is rare. However, TES is often associated with pelvic endometriosis with an incidence varying from 18% to 84%.^[2,6,13,21,22]^ In this study, 87.5% had concomitant TES and pelvic endometriosis. Studies have suggested that pelvic endometriosis usually occurs about 5 years earlier than onset of thoracic manifestation.^[13]^

Of the six patients who wished to become pregnant, only one had children, with the remaining 83.3% being infertile. The association between endometriosis and infertility has been well documented, with 30% - 50% of patients with endometriosis estimated to be infertile, while about 20% - 50% of infertile women are said to have endometriosis.^[23,24]^ The presence of endometrioma in the pelvis, with resultant adhesions, has been coined the ‘pelvic factor’. The distortion that arises causes tubo-ovarian dis-co-ordination, and affects tube patency.^[23,24]^ Other possible mechanisms include endocrine and ovulatory abnormalities, altered peritoneal function and altered endometrial hormonal and cell-mediated function.^[24]^

Diagnostic criteria in the establishment of thoracic endometriosis include clinical and histological factors, and the use of a biomarker. The establishment of cyclical symptoms in temporal relationship with menstruation, as seen in all patients in this study, is pivotal to diagnosis.^[6,16,21]^ However, a confirmatory diagnosis is established when endometrial stroma and glands are identified histologically, as was seen in 50% of our patients.^[2,6]^ Obtaining tissue for histological diagnosis may not always be feasible, and pleural fluid or bronchial lavage cytology is often negative. The identification of endometriotic lesions by VATS or bronchoscopy may be easier during menstruation. Bronchoscopy performed within 2 days of onset of menses may improve localisation, especially in patients with CHp.^[3,5]^ The appearances of lesions on radiological imaging techniques are nonspecific.^[2,5,10]^ Focal areas of consolidation, ill-defined opacities or bullous disease on the lung, or hypo-attenuated areas on the diaphragm seen on chest computed tomography scans are not pathognomonic, and the sensitivity on magnetic resonant imaging may be superior.^[2,3,5,10]^ The biomarker serum CA-125 is now used to improve the diagnosis of endometriosis. However, an elevated serum or pleural fluid level is nonspecific, as it is associated with any process causing irritation of mesothelial cells.^[3,10]^ We have only recently included this in the investigation protocol for our patients, and two of the three who had the test showed an elevated level.

Two patients from our study did not require surgical intervention. CSE in patient 7 resolved on intranasal oxygen supplementation while she was nursed in semi-Fowler’s position. CPT in patient 8 was mild and allowed for reabsorption. Of the remaining 6 patients, CTTD was the initial line of treatment in order to relieve raised intrapleural pressure. Two patients (1 and 3) had diagnostic VATS, but since our centre is not equipped for therapeutic VATS, parietal pleurectomy could not be conducted. Patient 2 did not achieve lung re-expansion for chemical pleurodesis and declined thoracotomy for parietal pleurectomy. She had a modified ambulatory home drainage system instituted. The other five patients all had chemical pleurodesis; in one (20%) this was successful at first application, another one (20%) at second application, and two (40%) failed and proceeded to have thoracotomy and parietal pleurectomy. One patient with recurrence declined further intervention. Our observation that chemical pleurodesis alone has a poor success rate in patients with CPE is supported by the literature.^[9,10,16]^ This is expected, as continuous activity from cyclical proliferation of endometrial implants, and also migration through patent diaphragmatic defects, predisposes to recurrence.

We recorded no mortality, however, and patients were referred to the gynaecologist for hormonal therapy and further management.

Despite individual case requirements, we propose a systematic approach to the management of TES, by a multidisciplinary team, consisting of a gynaecologist, cardiothoracic surgeon, pulmonologist, histopathologist, radiologist and anaesthesiologist.^[3,11]^ A high index of suspicion is needed on clinical assessment and radiologic evaluation. Initial supportive oxygen, observation and rest are instituted for small collections. Patients in respiratory distress need immediate relief by either thoracocentesis or tube thoracostomy, with fluid specimens obtained for microscopy, Ziehl Neelsen stain, chemistry, cytology and CA-125 assay. When available, VATS should be employed early. This is currently the gold standard, as both diagnosis and treatment (including resection of implants, closure of diaphragmatic fenestrations and pleurectomy with abrasive pleurodesis) can be effected with the attendant benefit of minimal access.^[3,5,11,21]^ Combined VATS and video-assisted laparoscopy is recommended by some researchers ^[10]^ Conventional thoracotomy should be utilised where VATS is unavailable, in cases of recurrence after VATS or failure of the minimally invasive technique. Hormone therapy using gonadotrophin-releasing hormone analogue is recommended in the immediate postoperative period and for 6 - 12 months afterwards.^[2,6,10]^

Two limitations of this study are its retrospective nature, and the small sample size. We have therefore constituted an endometriosis study group to collate prospectively collected data for future presentation.

## Conclusion

TES remains an uncommon condition despite being the most common extrapelvic manifestation of endometriosis. Its association with pelvic presentation is further strengthened by this study. There is some variability in the modes of manifestation of TES. Recurrent chest symptoms in a woman of childbearing age with a history of infertility should raise a high index of suspicion. Despite most literature reporting CPT as the most common TES manifestation, we found CPE to account for the majority of cases in our study. We also present a rare manifestation of CSE. Being a developing country with paucity of facilities, the utilisation of VATS as a treatment option is limited in Nigeria. Chemical pleurodesis and conventional thoracotomy with parietal pleurectomy are therefore the most common intervention modes. However, we found that chemical pleurodesis was generally unsuccessful in patients with TES; therefore, performing thoracotomy without attempting chemical pleurodesis may be a more beneficial in absence of VATS.
